# Comparative Optimization of Culture Media for Enhanced β‐Glucan Production by *Limosilactobacillus fermentum* SH2 (MW897962) and Its Bioactivity Assessment

**DOI:** 10.1002/fsn3.70960

**Published:** 2025-09-19

**Authors:** Tarek Gamal Abedelmaksoud, Shatha A. Allaith, Ammar B. Altemimi, Mohamed E. Abdel‐aziz, Zaid Akram Thabit, Mohammad Ali Hesarinejad, Reda Mahgoub Mohamed

**Affiliations:** ^1^ Food Science Department Faculty of Agriculture, Cairo University Giza Egypt; ^2^ Field Crops Department Faculty of Agriculture, Karbala University Karbala Iraq; ^3^ Department of Food Science College of Agriculture, University of Basrah Basrah Iraq; ^4^ Biotechnology Research Center, Environmental Biotech Department Al‐Nahrain University Baghdad Iraq; ^5^ Department of Food Sensory and Cognitive Science Research Institute of Food Science and Technology (RIFST) Mashhad Iran

**Keywords:** β‐glucan production, bioactivity, *Limosilactobacillus fermentum*, RSM

## Abstract

This study optimized β‐glucan production from the local isolate *Limosilactobacillus fermentum* SH2 (MW897962) and evaluated its bioactivity. Using Response Surface Methodology (RSM) with a Central Composite Design (CCD), exopolysaccharide (EPS) production was optimized in Semi‐Defined Media (SDM) and Chemically Defined Media (CDM). Predicted optimal β‐glucan yields were 2.42 g/100 mL for PYE and 29.65 g/100 mL for molasses, with molasses doubling EPS production under optimal conditions. The actual EPS yield (950.65 mg/100 mL) exceeded the predicted yield (931.54 mg/100 mL), while cell mass (129.95 mg/100 mL) was slightly lower than predicted (131 mg/100 mL). High R^2^ values for β‐glucan production (91.3%) and cell mass (90.2%) validated the optimization model. The β‐glucan demonstrated potent bioactivity, including 91% DPPH radical scavenging at 200 μg/mL. Cytotoxicity against Caco‐2 colorectal cancer cells showed 85.64% viability at 100 μg/mL, comparable to the β‐glucan standard (84.89%). At 1.0 and 10 μg/mL, β‐glucan from LAB showed greater inhibition of Caco‐2 cell proliferation than the standard. Antibacterial activity against four pathogens revealed the highest inhibition zone (14.00 mm) at 100 mg/mL against *Staphylococcus aureus* ATCC. This study highlighted the potential of *L. fermentum* SH2 as a cost‐effective source of β‐glucan with significant antioxidant, antitumor, and antibacterial properties.

## Graphical abstract

The study optimized β‐glucan production from *Limosilactobacillus fermentum* SH2 using Response Surface Methodology. Molasses‐based media significantly enhanced exopolysaccharide yield. The β‐glucan exhibited strong antioxidant (91% DPPH inhibition), anticancer (85.64% Caco‐2 cell viability), and antibacterial (14 mm zone against *S. aureus* ) activities. These findings confirm the strain's potential as a cost‐effective source of bioactive β‐glucan.

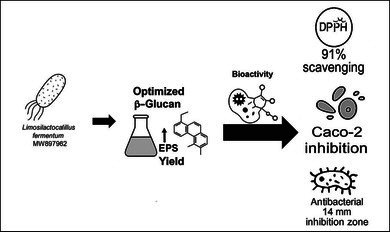

## 1 Introduction

Lactic acid bacteria (LAB), particularly *Limosilactobacillus fermentum*, are widely recognized for their prevalence in fermented foods and the gastrointestinal microbiota of humans and animals. Classified as Generally Recognized as Safe (GRAS), *L. fermentum* plays a pivotal role in the fermentation of diverse food substrates, including vegetables, cereals, dairy products, and meats (Ale, Rojas, et al. 2020; Ale, Batistela, et al. 2020; Lefeber et al. 2011). Its adaptability and probiotic properties have attracted considerable scientific interest, notably due to its capacity to synthesize exopolysaccharides (EPSs)—high‐molecular‐weight polysaccharides that function as protective barriers and bioactive agents. EPSs produced by LAB such as *L. fermentum* have gained significant attention not only for their ecological functions in microbial stress tolerance but also for their technological applications and health‐promoting potential. These biopolymers can be present as tightly bound capsules or as components of biofilms, enhancing microbial adhesion, resistance to environmental stressors, and survival under fluctuating pH and temperature conditions (Kleerebezem et al. 2010). Importantly, EPSs are classified within the broader category of postbiotics—non‐viable microbial derivatives that confer health benefits to the host, including enzymes, peptides, and polysaccharides (Aguilar‐Toala et al. 2018). Among EPS variants, β‐glucans—a class of homopolysaccharides frequently synthesized by LAB—offer multifaceted advantages. These compounds contribute to improved food texture and stability, enhanced water retention, and serve as prebiotics exhibiting antioxidant, immunomodulatory, antitumor, and cholesterol‐lowering effects (Wang et al. 2017; Imran et al. 2016). Despite these benefits, a critical limitation hindering the industrial exploitation of EPS is the typically low yield observed in many LAB strains, which constrains their commercial viability in food and pharmaceutical sectors (Ryan, Burdíková, et al. 2015; Ryan, Ross, et al. 2015). The composition of the growth medium is a decisive factor influencing EPS biosynthesis. Research indicates that semi‐defined media (SDM) and chemically defined media (CDM) yield more reproducible and enhanced EPS production compared to complex media like MRS broth, whose undefined components may obscure production outcomes (Ale, Rojas, et al. 2020; Ale, Batistela, et al. 2020; Kimmel et al. 1998). Notably, the incorporation of sucrose as a carbon source alongside nitrogen sources such as bacto casitone and ammonium citrate has proven optimal for β‐glucan biosynthesis in *L. fermentum* strains. For example, the EPS produced by *L. fermentum* Lf2 comprises a high‐molecular‐weight (1 → 3)‐β‐d‐glucan and a medium‐molecular‐weight heteroglycan, both exhibiting promising functional attributes (Vitlic et al. 2019; Ale, Rojas, et al. 2020; Ale, Batistela, et al. 2020).

The integration of β‐glucan‐producing probiotic bacteria into functional foods is gaining momentum, owing to the synergistic health benefits conferred by the probiotics and the β‐glucan polysaccharides. This combination enhances digestive health, modulates the microbiome, boosts immune responses, lowers cholesterol, and regulates glycemic levels (Latif et al. 2023). Incorporation of such strains into fermented dairy products (Lee et al. 2020; Fernandez and Marette 2017), fortified beverages (Mousanejadi et al. 2023; Kardooni et al. 2023; Behbahani et al. 2024; Echresh et al. 2025), and plant‐based alternatives increases nutritional value and provides dual health functions without reliance on synthetic additives (da Cruz Nascimento et al. 2024). Moreover, the bioavailability and stability of β‐glucan within these food matrices enhance their functional efficacy, supporting contemporary trends toward preventative and natural nutrition. In parallel, the pursuit of economically feasible substrates such as molasses for commercial EPS production is intensifying. However, process optimization remains critical. Traditional one‐factor‐at‐a‐time approaches are inefficient and provide limited insight, whereas statistical tools like Response Surface Methodology (RSM) enable simultaneous multi‐variable optimization, facilitating predictive modeling of EPS yield. Previous studies have reported 2 to 3‐fold increases in EPS production following RSM‐guided optimization of culture conditions (Deepak, Ramachandran, et al. 2016; Deepak, Kumar Pandian, et al. 2016; Wang et al. 2017). Despite these advances, comparative evaluations of SDM and CDM effects on β‐glucan production by *L. fermentum* remain scarce. Existing research predominantly addresses EPS structural characterization or optimization within a single medium, with limited focus on comparative bioactivity assessment.

This study aims to fill this research gap by systematically investigating the impact of two defined media types—semi‐defined medium (SDM) and chemically defined medium (CDM)—on the production and biofunctional properties of β‐glucan synthesized by a local isolate, *Limosilactobacillus fermentum* SH2 (MW897962), under optimized fermentation conditions. Specifically, the study objectives are to: maximize β‐glucan yield in both SDM and CDM using Response Surface Methodology for process optimization; characterize and compare the structural features and bioactivity profiles of β‐glucans produced in each medium; evaluate the feasibility and implications of utilizing defined media for scalable industrial β‐glucan production. By integrating media optimization with comprehensive bioactivity assessment, this work seeks to advance the fundamental understanding of EPS biosynthesis in *L. fermentum* and inform practical strategies for developing cost‐effective, high‐value functional food ingredients.

## 2 Materials and Methods

### 2.1 Chemicals

Acetone (Applichem, Indonesia), MRS broth (HiMedia, India; BioLab, Italy; BioLab, Hungary), Agar agar (Oxoid, UK), Ethanol 96% (Applichem, Indonesia), Anaerogene kits (ThermoFisher, UK), Methylene blue (Sigma, Denmark), Phenol (Merck, Germany), Sulfuric acid 99% (Merck, Germany), Trichloroacetic acid (TCA) (Advent, India), Peptone (Meron, India), Tri‐ammonium citrate 97% (SDFCL, India), Glucose (Qualikems, India), Sucrose (Qualikems, India), Yeast extract (Qualikems, India), Copper sulphate crystals (Fluka, UK), Potassium sodium tartrate (Fluka, UK), Sodium hydroxide pellets (SDFCL, India), β‐glucan standard (Now, USA), Cane molasses (El‐Hawamdia, Egypt), 2,2‐diphenyl‐1‐picrylhydrazyl (DPPH) (Sigma‐Aldrich, USA), Muller Hinton Agar (HiMedia, India), Brain Heart Infusion (BHI) broth (Oxoid, UK), de Man, Rogosa, and Sharpe (MRS) broth (BioLab, Hungary) were used.

### 2.2 Lactic Acid Bacteria and Growth Conditions

Based on our previous study, Allaith et al. (2022) described preliminary screening for a selection of one isolate among four LAB isolates as well as studying many factors (carbon sources, and nitrogen sources) and many factors constant (Initial number of bacteria, pH, time of fermentation, and ammonium citrate). *Limosilactobacillus fermentum* SH2 (MW897962), as the superior local isolate in the production of EPS (β‐glucans) was selected. It was obtained from the laboratories of the Department of Food Science, Faculty of Agriculture, University of Cairo. The stock cultures of *Limosilactobacillus fermentum* SH2 have grown in MRS broth with glycerol (20% v/v) under frozen storage (−20°C). The strain was activated by transfer to a fresh medium of MRS broth and incubated at 37°C for 48 h. β‐glucan production by *L. fermentum* SH2 using the RSM was carried out by batch fermentation under the following optimum conditions: pH 7, temp. 30°C, incubation time 48 h.

### 2.3 Experimental Design

Response Surface Methodology (RSM) was employed to statistically optimize the medium composition for maximizing β‐glucan production by *Limosilactobacillus fermentum* SH2 (MW897962). A Central Composite Design (CCD) was implemented using Design‐Expert software (version 11.1.2.0, Stat‐Ease Inc., USA) to evaluate the effects of three independent variables: the concentration of peptone‐yeast extract mixture (*x*_1_, 1–4 g/100 mL), glucose (*x*_2_, 1–4 g/100 mL), and sucrose (*x*_3_, 6–12 g/100 mL) in semi‐defined medium (SDM). For the chemically defined medium (CDM), two variables were considered: *x*_1_ (peptone‐yeast extract, 1–4 g/100 mL) and *x*_2_ (molasses, 20–40 g/100 mL), replacing glucose and sucrose (Oleksy‐Sobczak and Klewicka 2020; Othman et al. 2018). The response variables were Y₁ (exopolysaccharide [EPS] production) and Y₂ (cell biomass), and 40 experimental runs were carried out. The basal medium components remained constant: 0.2% tri‐ammonium citrate (97% purity), pH 7.0, and a standardized inoculum concentration of 1.5 × 10^8^ CFU/mL based on the McFarland turbidity standard.

The experimental data were fitted to a second‐order polynomial model (Equations 1 and 2):

(1) $$\mathrm{Two}\;\text{variables}:Y=a_{0}+a_{1}x_{1}+a_{2}x_{2}+a_{12}x_{1}x_{2}+a_{11}{x_{1}}^{2}+a_{22}{x_{2}}^{2}$$

(2) $$\begin{array}{c} \text{Three variables}:Y=a_{0}+a_{1}x_{1}+a_{2}x_{2}+a_{3}x_{3}+a_{12}x_{1}x_{2}+a_{13}x_{1}x_{3}\\ \;+a_{23}x_{2}x_{3}+a_{11}{x_{1}}^{2}+a_{22}{x_{2}}^{2}+a_{33}{x_{3}}^{2} \end{array}$$

where *Y* represents the predicted response (EPS production or cell mass), *x*_1_, *x*_2_, and *x*_3_ are the coded independent variables, and the coefficients *a*_0_ (intercept), *a*_1–_ *a*_3_ (linear), *a*_12–_ *a*_23_ (interaction), and *a*_11–_ *a*_33_ (quadratic) were determined via regression analysis. Analysis of variance (ANOVA) was conducted to evaluate the significance of model terms and fit adequacy. Model performance was assessed using the coefficient of determination (*R* ^2^), adjusted *R* ^2^, and the Prediction Error Sum of Squares (PRESS). A strong model was indicated by high *R* ^2^ and adjusted *R* ^2^ values (> 0.90), low PRESS, and non‐significant lack‐of‐fit. Perturbation and contour plots were generated to illustrate factor effects and interactions within the experimental domain. Optimization was performed using the desirability function approach, targeting the simultaneous maximization of both EPS production and cell biomass, as described in prior studies (Abedelmaksoud et al. 2018).

### 2.4 Isolation and Quantification of EPS Under Optimal Conditions

Triplicate experimental units (40 experiments) for both SDM and CDM were conducted in 300 mL flasks containing a final volume of 100 mL with concentrations as specified in Tables 1 and 3. All flasks were sterilized at 121°C for 15 min at 15 psi. To prevent Maillard reactions, the saccharide solutions and cane molasses were sterilized separately (Oleksy‐Sobczak and Klewicka 2020; Othman et al. 2018). Approximately 10% (v/v) of the inoculum was prepared from *Limosilactobacillus fermentum* SH2, which was suspended in MRS broth and incubated at 37°C for 48 h. SDM and CDM media were inoculated with the bacterial culture under sterile conditions, then incubated at 30°C, 130 rpm for 48 h in a rotary shaker (Innova‐shaker, Thomas Scientific, USA). After incubation, the fermented cultures were heated at 100°C for 15 min to inactivate enzymes, then centrifuged (Thermo centrifuge, USA) at 12,000*g* for 20 min at 4°C to separate the cells from the supernatant. The cell mass was collected and dried in an oven at 60°C overnight. Two volumes of cold acetone (96%) were added to the supernatant and left overnight at 4°C to precipitate the exopolysaccharides (EPS), primarily β‐glucans. The EPS precipitate was collected by centrifugation (10,000*g*, 10 min, 4°C) and dissolved in water. An equal volume of 15% (w/v) trichloroacetic acid (TCA) was added and incubated for 2 h to precipitate proteins, which were then removed by centrifugation (12,000*g*, 20 min, 4°C). The supernatant was collected and dialyzed against distilled water using 12–14 kDa molecular weight cutoff (MWCO) membranes (USA) at 4°C for 3 days, with daily water changes. The purified EPS solution was freeze‐dried (CHRIST alpha 1–4 LSC plus, Germany), weighed (mg/100 mL), and stored at room temperature in a desiccator.

**TABLE 1:** Actual experimental data on the effect of glucose, sucrose, and peptone yeast extract (PYE) concentrations on exopolysaccharide (EPS) production and biomass of *L. fermentum* SH2 in a semi‐defined medium.

Sample no.	PYE (*x*_1_) g/100 mL	Glucose (*x*_2_) g/100 mL	Sucrose (*x*_3_) g/100 mL	EPS mg/100 mL	Cell mass mg/100 mL
1	4	1	6	170	12
2	2.5	2.5	9	137	45
3	2.5	2.5	9	135	41
4	2.5	2.5	9	136	43
5	4	1	12	51	100
6	2.5	2.5	9	130	44
7	4	4	6	56	110
8	1	1	12	25	41
9	4	1	12	51	105
10	5.02	2.5	9	133	9
11	2.5	2.5	5.45	113	60
12	1	4	6	47	60
13	2.5	2.5	14.05	105	160
14	2.5	5.02	9	81	90
15	1	1	6	228	11
16	1	4	12	116	30
17	2.5	2.5	9	136	39
18	4	1	6	170	10
19	4	4	12	63	50
20	2.5	2.5	9	129	43
21	2.5	2.5	9	135	38
22	1	1	6	221	13
23	4	4	12	63	50
24	2.5	5.02	9	80	95
25	4	4	6	56	50
26	2.5	0	9	113	33
27	2.5	2.5	14.05	105	106
28	2.5	2.5	9	131	43
29	1	4	6	47	62
30	5.02	2.5	9	132	10
31	2.5	2.5	3.95	110	70
32	2.5	2.5	9	139	37
33	2.5	2.5	9	136	44
34	1	4	12	116	30
35	0	2.5	9	143	15
36	2.5	2.5	9	128	46
37	1	1	12	25.5	40
38	2.5	2.5	9	136	41
39	2.5	0	9	112	27
40	0	2.5	9	140	19

### 2.5 Analysis of Molasses and EPS

#### 2.5.1 Analysis of Chemical Composition of Molasses

The clarification of molasses was carried out following the method described by Thai (2013). After clarification, total soluble solids (°Brix) were measured using a portable refractometer (Trans Instruments, Singapore). Total carbohydrate content (%) was determined according to DuBois et al. (1956), total protein content (%) was measured following Classics Lowry et al. (1951), and ash content (%) was analyzed according to the AOAC method (1990).

#### 2.5.2 Analysis of Chemical Composition of Freeze‐Dried EPS

The total carbohydrate content in the EPS extracts was determined using the phenol–sulfuric acid method, with glucose as the standard (DuBois et al. 1956). The total protein content was measured by the Folin–Lowry method, using bovine serum albumin as the standard (Lowry et al. 1951).

#### 2.5.3 β‐Glucan Extraction

*L. fermentum* SH2 was centrifuged at 12,000*g* for 20 min at 4°C following fermentation. To precipitate exopolysaccharides (EPS) as β‐glucans, two volumes of cold 96% acetone were added to the supernatant and incubated overnight at 4°C. The resulting precipitate was collected by centrifugation at 10,000*g* for 10 min at 4°C. The EPS was then dissolved in distilled water and dialyzed using 12–14 kDa molecular weight cutoff (MWCO) membranes (USA) with daily changes of distilled water over a period of 3 days at 4°C. Subsequently, the dialyzed EPS solution was freeze‐dried (CHRIST alpha 1–4 LSC plus, Osterode am Harz, Germany). The yield was determined as mg per 100 mL, and the dried EPS was stored in a desiccator at room temperature (Allaith et al. 2022).

#### 2.5.4 Determination of β‐Glucans by HPLC After Optimization

β‐glucans were quantitatively and qualitatively analyzed by HPLC using a Waters Alliance 2695 Separations Module equipped with a Benson polymeric Bp100 Ca column and a Waters 2410 Refractive Index Detector. The mobile phase was deionized water (DI H₂O), with a flow rate of 0.4 mL/min. The sample injection volume was 20 μL (30 mg/mL), and the column dimensions were 100 × 7.8 mm (Allaith et al. 2022).

### 2.6 The Bioactivity Characterization of β‐Glucan Production After Optimization

#### 2.6.1 Estimation of Antioxidant Activity

The DPPH (2,2‐diphenyl‐1‐picrylhydrazyl) assay was used to determine IC₅₀ values with some modifications according to Ali and El Said (2020) and Abd El Ghany et al. (2016). Approximately 0.25 g of β‐glucan extract was dissolved in 2.5 mL of distilled water to prepare a stock solution with a concentration of 100 mg/mL (100,000 μg/mL). From this stock solution, aliquots of different amounts (50, 75, 100, 150, and 200 μg) were taken and diluted to a final volume of 1 mL with distilled water to obtain the respective concentrations (μg/mL). Each sample was then mixed with 3 mL of DPPH solution (0.0019 g/100 mL absolute methanol). After incubation for 30 min at room temperature in the dark, the absorbance of the mixture was measured at 517 nm using a spectrophotometer. The free radical scavenging activity was expressed as % inhibition, calculated according to Equation (3):

(3) $$\text{Inhibition}\;\left(\%\right)=\left(A\right)\;\text{control}–\left(A\right)\;\text{sample}/\left(A\right)\;\text{control}\times 100$$

where *A* is the absorbance of the control and sample, respectively; Control is DPPH; Blank is absolute methanol.

#### 2.6.2 Estimated of the Cytotoxicity

##### 2.6.2.1 Cell Culture

Caco‐2 cells (colorectal cancer) were obtained from Nawah Scientific Inc. (Mokatam, Cairo, Egypt). The cells were maintained in RPMI medium supplemented with 100 μg/mL streptomycin, 100 units/mL penicillin, and 10% heat‐inactivated fetal bovine serum, and incubated in a humidified atmosphere containing 5% (v/v) CO₂ at 37°C.

##### 2.6.2.2 Cytotoxicity Assay

**FIGURE 1:**
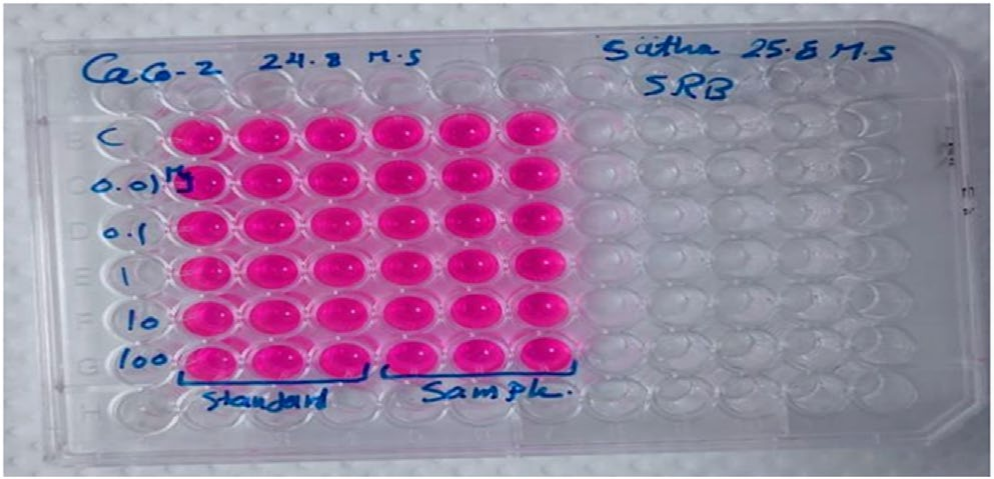
Tissue culture plate method to estimate the cytotoxicity of β‐glucan on the viability (%) of colorectal cancer (Caco‐2) cells.

Cell viability was assessed using the Sulforhodamine B (SRB) assay with appropriate controls: a blank (medium without cells), untreated control cells (without drug), and standard β‐glucan as a positive control. Aliquots of 100 μL of cell suspension containing 5 × 10^3^ cells were seeded into 96‐well plates and incubated in complete medium for 24 h. Subsequently, cells were treated with 100 μL aliquots of medium containing the test compounds: β‐glucan extracted from LAB (sample) and standard β‐glucan (positive control) at varying concentrations (0.01, 0.1, 1.0, 10, and 100 μg/mL). After 72 h of drug exposure, cells were fixed by replacing the medium with 150 μL of 10% trichloroacetic acid (TCA) and incubated at 4°C for 1 h. Following fixation, the TCA solution was removed, and the cells were washed five times with distilled water. Then, 70 μL of SRB solution (0.4% w/v) was added to each well and incubated in the dark at room temperature for 10 min. Plates were subsequently washed three times with 1% acetic acid and left to air‐dry overnight. To solubilize the protein‐bound SRB stain, 150 μL of 10 mM Tris base (TRIS) was added to each well. Absorbance was measured at 540 nm using a BMG LABTECH FLUOstar Omega microplate reader (Ortenberg, Germany) (Skehan et al. 1990; Allam et al. 2018) (Figure 1). The mean absorbance values for each drug concentration were calculated after automatic subtraction of the mean background absorbance. Cell viability (%) was calculated according to Equation (4).

(4) $$\text{The viability}\%=\mathrm{OD}\;\left(\text{treated cells}\right)/\mathrm{OD}\;\left(\text{control cells}\right)\ldots \ldots$$

In addition, the IC_50_ values, which refer to the concentrations of resveratrol that are necessary to achieve a 50% inhibition of cell growth, were computed.

#### 2.6.3 Estimation of the Antibacterial Activity

*Staphylococcus aureus* ATCC 25923 and *Pseudomonas aeruginosa* ATCC 9027 were obtained from the American Type Culture Collection (ATCC), Rockville, Maryland, USA. Enterococcus faecalis 90 and *Staphylococcus aureus* 112 were provided by the Food Science Department, Faculty of Agriculture, Cairo University, Giza, Egypt. The antibacterial activity of β‐glucan extracted from lactic acid bacteria (LAB) was assessed using the agar diffusion assay described by Bonev et al. (2008). Briefly, 0.1 mL of each pathogenic bacterial suspension (24 h old), adjusted to approximately 1.0 × 10^8^ CFU/mL, was spread evenly over the surface of Muller‐Hinton agar plates using an L‐shaped glass rod. The plates were allowed to dry for 15 min. Wells were then created in the agar using a sterile cork borer, and 50 μL of β‐glucan solutions at concentrations of 50 and 100 mg/mL (prepared by dissolving 0.25 g freeze‐dried β‐glucan in 2.5 mL distilled water for the stock solution) were added into the wells. Distilled water served as the negative control. The plates were incubated at 37°C for 24 h, after which the inhibition zones were measured in millimeters, subtracting the 5 mm well diameter. The assay was performed in duplicate to ensure reproducibility.

### 2.7 Statistical Analysis

The analysis of variance (ANOVA) XLSTAT software 2014 was used in three repetitions to assess the experimental results, which were presented as mean ± standard error of the mean. A *p*‐value ≤ 0.05 was used to determine the significance of sample mean differences.

## 3 Results and Discussion

### 3.1 *Limosilactobacillus fermentum* SH2 (MW897962) and Optimization Study

Based on preliminary screening conducted to select one isolate from four lactic acid bacteria (LAB) strains reported in our previous study by Allaith et al. (2022), the bacterial strain *Limosilactobacillus fermentum* SH2 (MW897962) was selected due to its superior production of exopolysaccharides (EPS) and β‐glucan, yielding 481 ± 1.00 mg/100 mL and 5.56% ± 0.01%, respectively. The preliminary EPS production was assessed in a semi‐defined medium (SDM) composed of 100 mL distilled water, 0.5% yeast extract, 1.0% peptone, 0.2% ammonium citrate, and 2.0% sucrose.

### 3.2 Optimization of EPS Production by *Limosilactobacillus fermentum* SH2

Exopolysaccharide (EPS) biosynthesis initiates intracellularly and culminates in extracellular secretion, influenced by a variety of environmental and nutritional factors. Despite extensive research on bacterial EPS, the mechanisms regulating its production are not yet fully understood (Sutherland 2001). Earlier efforts to optimize EPS production primarily employed conventional approaches lacking statistical rigor. In recent years, the application of advanced statistical methodologies—such as Central Composite Design (CCD), Plackett‐Burman, D‐optimal, fractional factorial, and Box–Behnken designs—has significantly improved the efficiency of optimization strategies (Ale, Rojas, et al. 2020; Ale, Batistela, et al. 2020; Oleksy‐Sobczak and Klewicka 2020; Othman et al. 2018). Carbon sources such as glucose, sucrose, and molasses play a pivotal role in EPS synthesis, given that sugars are fundamental constituents of EPS. Similarly, nitrogen sources like peptone and yeast extract have been shown to positively influence production yields (Zhao et al. 2020; Othman et al. 2018). In the present study, Response Surface Methodology (RSM) was employed to optimize the fermentation medium for enhancing β‐glucan production by *Limosilactobacillus fermentum* SH2 (MW897962). A Central Composite Design (CCD) was implemented using Design‐Expert software (version 11.1.2.0, Stat‐Ease Inc., USA) to evaluate the effects of three key independent variables: *x*_1_ (PYE, a 1:1 mixture of peptone and yeast extract), *x*_2_ (glucose or molasses, depending on the selected medium—synthetic defined medium [SDM] or complex defined medium [CDM]), and *x*_3_ (sucrose, included in SDM only). A total of 40 experimental runs were conducted, as detailed in Tables 1 and 3, to investigate the optimal conditions for maximizing EPS yield and cell biomass.

#### 3.2.1 Optimization of Semi‐Defined Media (SDM) for EPS and Biomass Production

Exopolysaccharide (EPS) production and biomass yield were optimized using response surface methodology (RSM) with a central composite design (CCD) comprising 40 experimental runs. The effects of three independent variables—PYE concentration (*x*_1_), glucose (*x*_2_), and sucrose (*x*_3_)—were systematically evaluated for their individual and interactive contributions to EPS synthesis and cell growth. The highest predicted desirability (0.424) was obtained at an optimal medium composition of 2.96 g/100 mL PYE (1:1 peptone: yeast extract), 3.46 g/100 mL glucose, and 12.00 g/100 mL sucrose. Under these conditions, EPS and biomass production reached 114.09 and 70.80 mg/100 mL, respectively (Table 4). Table 1 presents the complete design matrix and corresponding responses. The optimization results were consistent with prior findings. Zhao et al. (2020) reported a 2.9‐fold increase in EPS production by *Weissella confusa* XG‐3 under optimized conditions (80.1 g/L sucrose, pH 5.8, 3.7 g/L sodium acetate). Similarly, Ale, Rojas, et al. (2020), Ale, Batistela, et al. (2020) enhanced EPS yields using a semi‐defined medium optimized at pH 6.5, while Oleksy‐Sobczak and Klewicka (2020) reported more than a 13‐fold increase in EPS production by *Lactobacillus rhamnosus* strains using a Plackett–Burman design. In the present study, sucrose concentration was identified as the most influential factor for EPS production. For instance, the lowest EPS yield (25.5 mg/100 mL) was recorded at minimal PYE and glucose concentrations (1.0 g/100 mL each), despite the presence of 12.0 g/100 mL sucrose. Conversely, the highest EPS yield (224.5 mg/100 mL) was obtained with low PYE and glucose (1.0 g/100 mL) and a moderate sucrose level (6.0 g/100 mL), although the cell mass was substantially reduced (12.0 mg/100 mL), suggesting a dissociation between EPS synthesis and microbial biomass under certain conditions.

Notably, substantial EPS levels were produced even in the absence of PYE, indicating its non‐essentiality for EPS biosynthesis but potential role in product stabilization. While glucose had a limited role in EPS production, it significantly contributed to cell mass. For example, media containing 2.5 g/100 mL PYE and 5.02 g/100 mL glucose yielded 80.5 mg/100 mL EPS and 92.5 mg/100 mL biomass. This observation aligns with previous reports highlighting sucrose and fructose as more favorable carbon sources for EPS production by *L. rhamnosus* (Oleksy‐Sobczak and Klewicka 2020). Sucrose concentrations between 3.95 and 14.05 g/100 mL promoted EPS yields ranging from 105 to 134.36 mg/100 mL; however, excessive sucrose led to osmotic stress, adversely affecting both EPS output and biomass. These outcomes are in agreement with findings from *Leuconostoc* spp. and *L. confusus* TISTR 1498, where elevated sucrose levels resulted in production inhibition (Shaileshkumar and Lele 2010; Manochai et al. 2014). Allaith et al. (2022) similarly reported an inverse relationship between EPS yield and biomass, attributing high EPS levels to the expression of glucosyltransferase (gtf) genes rather than microbial proliferation. Analysis of variance (ANOVA) for the CCD model (Table 2) confirmed its statistical significance for both responses (*p* < 0.0001), with *R* ^2^ values of 0.8266 and 0.8051, and adjusted *R* ^2^ values of 0.7746 and 0.7467 for EPS and biomass, respectively. These results validate the predictive reliability of the quadratic models, consistent with previous studies (Manochai et al. 2014; Ale, Rojas, et al. 2020; Ale, Batistela, et al. 2020). Among the tested factors, glucose (*x*_2_) and sucrose (*x*_3_) had highly significant effects (*p* < 0.01) on both EPS and cell mass, while PYE (*x*_1_) significantly influenced biomass only (*p* = 0.0492). Regression coefficients with *p*‐values below 0.05 reinforced the significance of these variables in the model. The second‐order polynomial equations describing the responses are as follows:

(5) $$\begin{array}{c} \mathrm{EPS}\;\left(\mathrm{mg}/100\;\mathrm{mL}\right)=133.55-6.41x_{1}-17.67x_{2}-18.50x_{3}\\ \;-1.94x_{1}x_{2}+2.31x_{1}x_{3}+49.31x_{2}x_{3}\\ \;-2.55{x_{1}}^{2}-17.12{x_{2}}^{2}-12.31{x_{3}}^{2} \end{array}$$

(6) $$\begin{array}{c} \text{Cell Mass}\;\left(\mathrm{mg}/100\;\mathrm{mL}\right)=42.60+6.55x_{1}+11.75x_{2}+11.38x_{3}\\ \;-2.81x_{1}x_{2}+8.06x_{1}x_{3}-22.56x_{2}x_{3}\\ \;-12.99{x_{1}}^{2}+4.21{x_{2}}^{2}+19.30{x_{3}}^{2} \end{array}$$

**FIGURE 2:**
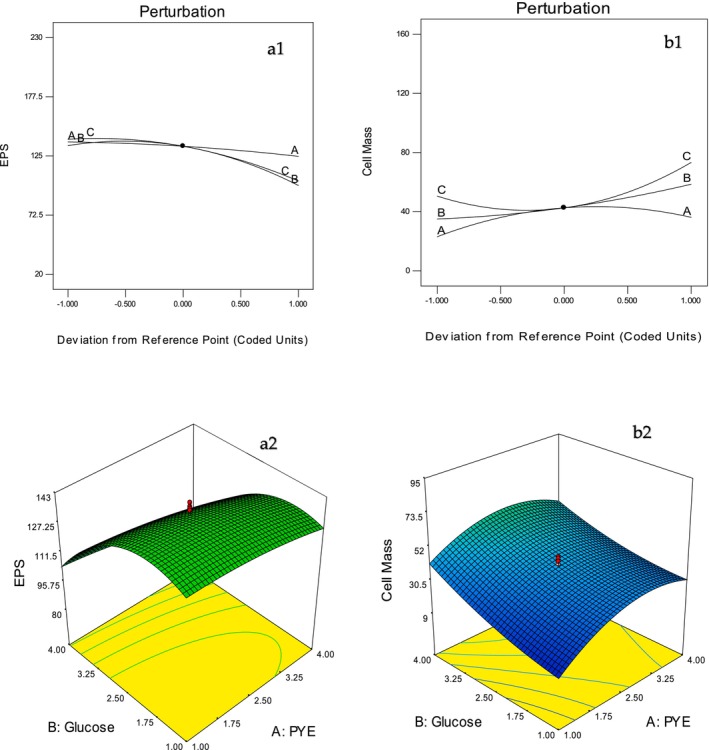
Perturbation graph and 3D response surface plots for EPS production mg/100 mL (*a*_1_ and *a*_2_) and cell mass mg/100 mL (b1 and b2) using CCD and semi‐defined media (SDM). *PYE concentration (A = *x*_1_), glucose (B = *x*_2_), and sucrose (C = *x*_3_).

Using Design Expert software, three‐dimensional response surface and contour plots (Figure 2) visualized the effects and interactions of the variables. Elliptical contour plots indicated significant interactions, particularly between sucrose and glucose concentrations. The predicted optimal levels (2.96 g/100 mL PYE, 3.46 g/100 mL glucose, and 12.00 g/100 mL sucrose) yielded 114.09 mg/100 mL EPS and 70.80 mg/100 mL biomass, which were in close agreement with experimental values (117.25 mg/100 mL EPS and 69.59 mg/100 mL biomass), further confirming the validity of the model (Table 4). These results are consistent with previous findings (Zhao et al. 2020; Almalki 2020) that underscore the efficacy of RSM in optimizing microbial metabolite production.

**TABLE 2:** Analysis of variance for EPS production and cell mass of *L. fermentum* SH2 using CCD design.

Source	DF	EPS, mg/100 mL				Cell mass, mg/100 mL			
		SS	MS	*F*‐value	*p*‐value	SS	MS	*F*‐value	*p*‐value
Model	9	70165.32	7796.15	15.89	< 0.0001** significant	34446.39	3827.38	13.77	< 0.0001 Significant
*X*_1_	1	1117.40	1117.40	2.28	0.1417**	1168.19	1168.19	4.20	0.0492*
*X*_2_	1	8499.00	8499.00	17.32	0.0002	3757.37	3757.37	13.52	0.0009
*X*_3_	1	8753.50	8753.50	17.84	0.0002	3309.60	3309.60	11.91	0.0017
*X*_1_ *x*_2_	1	60.06	60.06	0.12	0.7289	126.56	126.56	0.46	0.5050
*X*_1_ *x*_3_	1	85.56	85.56	0.17	0.6792	1040.06	1040.06	3.74	0.0625
*X*_2_ *x*_3_	1	38907.56	38907.56	79.30	< 0.0001	8145.06	8145.06	29.31	< 0.0001
*X*_1_^2^	1	185.87	185.87	0.38	0.5429	4818.45	4818.45	17.34	0.0002
*X*_2_^2^	1	8377.19	8377.19	17.07	0.0003	505.98	505.98	1.82	0.1873
*X*_3_^2^	1	3743.55	3743.55	7.63	0.0097	9205.92	9205.92	33.12	< 0.0001
Residual	30	14718.58	490.62			8337.59	277.92		
Lack of fit	6	14551.08	2425.18	347.49	< 0.0001	4934.09	822.35	5.80	0.0008
Pure error	24	167.50	6.98			3403.50	141.81		
Cor total	39	84883.90				42783.98			
*R* ^2^		0.8266				0.8051			
Adj‐*R* ^2^		0.7746				0.7467			
Pred‐*R* ^2^		0.6208				0.5720			
C.V. %		19.79				33.83			
PRESS		32191.24				18313.28			

Abbreviations: DF, degree of freedom; EPS, exopolysaccharide; MS, mean square; SS, sum of square.

* *p* < 0.05: indicated significant differences.

** *p* < 0.01: indicated extremely significant differences; *x*_1_: PYE (1:1 mixture from peptone: yeast extract); *x*_2_: Glucose; *x*_3_: Sucrose.

#### 3.2.2 Optimization of Chemically Defined Medium (CDM) for EPS and Biomass Production

The optimization of exopolysaccharide (EPS) and biomass production by *Lactobacillus fermentum* SH2 was carried out using Response Surface Methodology (RSM) based on a Central Composite Design (CCD). Two independent variables—PYE (1:1, peptone: yeast extract, *x*_1_) and molasses (*x*_2_)—were evaluated for their individual and interactive effects on EPS yield and cell mass (Table 3). A total of 40 experimental runs were designed by varying the concentrations of *x*_1_ and *x*_2_ in chemically defined medium (CDM). At the predicted optimal point (*x*_1_ = 2.42 g/100 mL, *x*_2_ = 29.65 g/100 mL), the model estimated maximum EPS production of 931.54 mg/100 mL and cell mass of 131 mg/100 mL, with a desirability score of 0.748 (Table 4). In contrast, the lowest EPS yield (~202.5 mg/100 mL) and biomass (114 mg/100 mL) occurred in runs lacking PYE (*x*_1_ = 0), indicating the critical role of nitrogen sources in supporting EPS matrix stability and cell proliferation. The highest EPS (958–1277.5 mg/100 mL) and biomass levels (137.59–268.5 mg/100 mL) were achieved at *x*_1_ = 2.5 g/100 mL and *x*_2_ ranging from 30 to 46.82 g/100 mL. These results demonstrated that increasing *x*_2_ while maintaining *x*_1_ at an optimal level significantly enhanced EPS production. For instance, molasses concentrations of 13.33, 30, and 46.82 g/100 mL yielded EPS levels of 455.9, 958, and 1277.5 mg/100 mL, respectively. Similarly, intermediate concentrations of both variables (e.g., *x*_1_ = 1 g/100 mL, *x*_2_ = 20–40 g/100 mL) produced moderate EPS and biomass yields, confirming a synergistic interaction between carbon and nitrogen sources. A combination of *x*_1_ = 4 g/100 mL and *x*_2_ = 40 g/100 mL resulted in 999.95 mg/100 mL EPS and 221.25 mg/100 mL biomass. However, further increasing *x*_1_ to 5.02 g/100 mL (samples 10 and 30) did not yield higher EPS levels, which plateaued around 415.5 mg/100 mL, indicating a threshold effect for PYE supplementation. Analysis of variance (ANOVA) revealed that the model was statistically significant (*p* < 0.0001) for both EPS and biomass responses, with high coefficients of determination (*R* ^2^ = 0.9130 and 0.9022, adjusted *R* ^2^ = 0.9002 and 0.8878, respectively), confirming the model's suitability for prediction. The polynomial regression equations generated (Equations 7 and 8) for EPS and biomass yield, respectively, were (Table 5):

(7) $$\begin{array}{c} \mathrm{EPS}\;\left(\mathrm{mg}/100\;\mathrm{mL}\right)=944.97+143.37x_{1}+163.25x_{2}+100.69x_{1}x_{2}\\ \;-250.79{x_{1}}^{2}-51.03{x_{2}}^{2} \end{array}$$

(8) $$\begin{array}{c} \text{Biomass}\;\left(\mathrm{mg}/100\;\mathrm{mL}\right)=134.63+32.18x_{1}+32.61x_{2}\\ \;+19.22x_{1}x_{2}+3.75{x_{1}}^{2}+15.43{x_{2}}^{2} \end{array}$$

**FIGURE 3:**
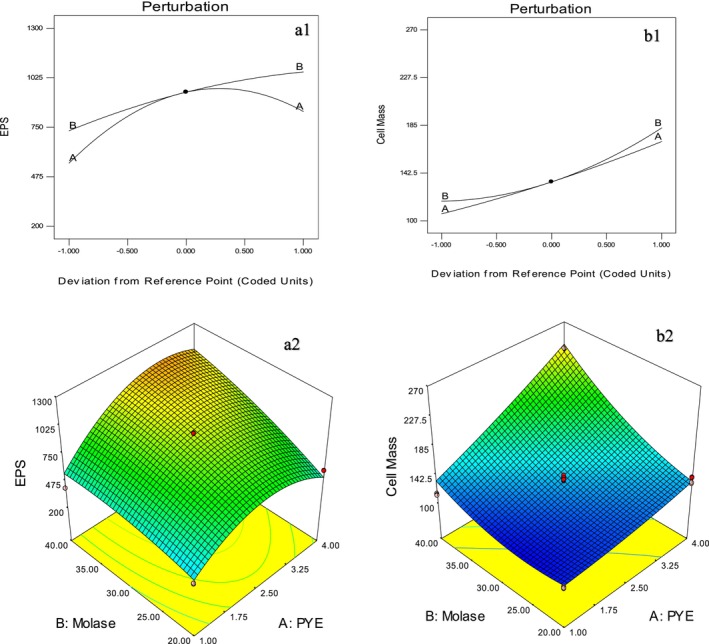
Perturbation graph and 3D response surface plots for EPS production mg/100 mL (*a*_1_, *a*_2_) and cell mass mg/100 mL (b1, b2) using CCD and chemical‐defined media (CDM): *PYE concentration (A = *x*_1_), and molasses (B = *x*_2_).

These models effectively captured the response patterns, as validated by the high congruence between predicted and observed values. Response surface and contour plots (Figure 3) were generated using Design‐Expert software to visualize the effects of *x*_1_ and *x*_2_ on EPS and biomass production. The elliptical shape of the contour plots indicated significant interaction between the variables. The actual maximum EPS (950.65 mg/100 mL) and biomass (129.95 mg/100 mL) values were consistent with model predictions, verifying its predictive capability. Statistical analysis also confirmed the significance of both *x*_1_ and *x*_2_ on EPS and biomass (*p* < 0.0001). These findings emphasize the potential of molasses as a cost‐effective carbon source for industrial EPS production by lactic acid bacteria (LAB), particularly in CDM compared to semi‐defined media (SDM). As reported by Othman et al. (2018), molasses supplementation significantly enhanced EPS and biomass production by *Lactobacillus plantarum* ATCC 8014. Similar outcomes were observed by Almalki (2020), who optimized EPS synthesis by *Lactococcus lactis* using CCD, and Manochai et al. (2014), who demonstrated a two‐fold EPS increase in sugarcane juice medium compared to MRS. Further support comes from Zhao et al. (2020), who reported regression coefficients (*R* ^2^ = 0.8011; adjusted *R* ^2^ = 0.6441) in EPS optimization by *Weissella confusa* XG‐3. In addition, Garai‐Ibabe et al. (2010) found *R* ^2^ values of 0.792, 0.933, and 0.952 for EPS production by *Pediococcus parvulus* CUPV1, CUPV22, and *Lactobacillus suebicus* CUPV221, respectively, validating the robustness of CCD for modeling microbial EPS synthesis. In conclusion, the integration of PYE (*x*_1_) and molasses (*x*_2_) in CDM significantly enhanced EPS and biomass production by *L. fermentum* SH2. The optimized process provides a basis for scalable, cost‐effective EPS production using accessible carbon sources for industrial and functional food applications.

**TABLE 3:** Actual experimental data on the effect of PYE (*x*_1_) and molasses (*x*_2_) on EPS and biomass production by *L. fermentum* SH2 in CDM.

Sample no.	PYE (*x*_1_) g/100 mL	Molasses (*x*_2_) g/100 mL	EPS mg/100 mL	Cell mass mg/100 mL
1	4	20	589	140
2	2.5	30	959	143
3	2.5	30	958	136
4	2.5	30	960	136.5
5	4	20	588	132
6	2.5	30	957	135.5
7	4	40	1001	230
8	1	20	400	104
9	4	20	587	139
10	5.023	30	416	206
11	2.5	30	959	140
12	1	40	406	114
13	2.5	30	956	134
14	2.5	46.82	1280	270
15	1	20	399	106
16	1	40	407	112
17	2.5	30	961	140
18	4	20	590	130
19	4	40	999.8	231
20	2.5	30	955	135
21	2.5	30	956.5	136
22	1	20	398.8	103
23	4	40	1000	225
24	2.5	46.82	1275	267
25	4	40	999	199
26	2.5	13.33	456	117
27	2.5	30	954	141.5
28	2.5	30	955.5	136
29	1	40	405	113
30	5.02	30	415	210
31	2.5	30	959.5	137
32	2.5	30	953.5	139
33	2.5	30	960.5	138
34	1	40	404.5	115
35	0	30	205	112.5
36	2.5	30	961.5	138
37	1	20	389.9	104.5
38	2.5	30	962	136
39	2.5	13.33	455.8	116
40	0	30	200	111.5

**TABLE 4:** The predicted and actual values for EPS and cell mass produced by *L. fermentum* SH2 for both media under optimal conditions.

Treatment	Predicted values		Actual values	
	EPS mg/100 mL	Cell mass mg/100 mL	EPS mg/100 mL	Cell mass mg/100 mL
SH2 in SDM	114.091	70.7955	117.25	69.592
SH2 in CDM	931.54	131	950.65	129.95

Abbreviations: Actual values, those were given by the actual results under this study based on the predicted factors; CDM, Chemical‐define media; Predicted values, those were given by experimental design under this study; SDM, Semi‐define media; SH2, *L. fermentum* .

**TABLE 5:** Analysis of variance for EPS production and cell mass of *L. fermentum* SH2 using CCD design.

Source	DF	EPS, g/100 mL				Cell mass, g/100 mL			
		SS	MS	*F*‐value	*p*‐value	SS	MS	*F*‐value	*p*‐value
Model	5	3.258E+006	6.516E+005	71.34	< 0.0001**	70287.62	14057.52	125.78	< 0.0001**
*x*_1_	1	5.593E+005	5.593E+005	61.24	< 0.0001**	28182.49	28182.49	129.12	< 0.0001**
*x*_2_	1	7.251E+005	7.251E+005	79.40	< 0.0001**	28929.16	28929.16	26.38	< 0.0001**
*x*_1_ *x*_2_	1	1.622E+005	1.622E+005	17.76	0.0002	5909.77	5909.77	1.80	0.1890
*x*_1_^2^	1	1.805E+006	1.805E+006	197.64	< 0.0001	402.50	402.50	30.48	< 0.0001
*x*_2_^2^	1	74724.50	74724.50	8.18	0.0072	6828.49	6828.49	125.78	< 0.0001
Residual	34	3.105E+005	9133.04			7617.86	224.05		
Lack of fit	3	3.103E+005	1.034E+005	15028.41	< 0.0001	6745.06	2248.35	79.86	< 0.0001
Pure error	31	213.36	6.88			872.80	28.15		
Cor total	39	3.568E+006				77905.47			
*R* ^2^		0.9130				0.9022			
Adj‐*R* ^2^		0.9002				0.8878			
Pred‐*R* ^2^		0.8473				0.8337			
C.V. %		12.92				10.13			
PRESS		5.450E+005				12954.74			

Abbreviations: DF, degree of freedom; EPS, exopolysaccharide; MS, mean square; SS, sum of square.

** *p* < 0.01: indicated extremely significant differences; *x*_1_: PYE (1:1 mixture from peptone: yeast extract); *x*_2_: Molasses.

### 3.3 Chemical Composition of Molasses and EPS

#### 3.3.1 Total Soluble Solid, Total Carbohydrate, Total Protein of Molasses

Following the clarification process, the chemical composition of molasses was characterized. The total soluble solids measured 41°Brix, with total carbohydrates at 5.6% ± 1.41%, total protein at 5.5% ± 0.28%, and ash content at 4.03% ± 0.01%. The relatively low ash content observed compared to previous findings (Othman et al. 2018) is likely due to the clarification step, which effectively reduced the mineral content. Cane molasses processed using a single clarification method typically yields sucrose concentrations around 69°Brix, while dual clarification methods yield slightly lower values (66.7°Brix–68.0°Brix) (Thai 2013). Protein content in clarified cane molasses has been reported within the range of 3.62%–6.08%, consistent with the present findings. Furthermore, total carbohydrates in molasses generally range from 57% to 71%, and ash content has been reported to average 13.1% (Palmonari et al. 2020). In comparison, the molasses used in this study showed a similar carbohydrate profile to that reported by Othman et al. (2018), who found 56% carbohydrates, 5.8% nitrogen, and a lower ash content of 3.3%. These results confirm that the clarification process significantly alters the mineral composition of molasses without drastically affecting its carbohydrate or protein content.

#### 3.3.2 Chemical Composition of EPS Under Optimal Conditions Compared to the Non‐Optimal Conditions

Table 6 presents the actual exopolysaccharide (EPS) yields (mg/100 mL) produced by *Lactobacillus fermentum* SH2 under optimal cultivation conditions using two media types: semi‐defined medium (SDM) and chemically defined medium (CDM). These values are compared with EPS production under non‐optimized conditions (designated B6), as previously reported by Allaith et al. (2022). Among all treatments, SH2 cultured in CDM exhibited the highest EPS yield (950.65 ± 3.00 mg/100 mL), followed by the non‐optimized condition (481 ± 1.00 mg/100 mL), while the lowest yield was observed in SDM (117.25 ± 1.00 mg/100 mL). EPS production by lactic acid bacteria (LAB) under non‐optimized conditions is typically variable and often limited, ranging between 0.045 and 0.350 g/L depending on the strain and medium composition (De Vuyst and Degeest 1999). The high EPS yield in CDM, nearly 1 g/L, exceeds previously reported values for this species, despite SH2 being a local isolate rather than a well‐characterized strain (Ale, Rojas, et al. 2020; Ale, Batistela, et al. 2020; Fukuda et al. 2010; Shi et al. 2014). This enhancement is likely attributable to the use of molasses as the sole carbon source, which has been shown to significantly enhance polysaccharide synthesis (Othman et al. 2018), in combination with peptone and yeast extract that support EPS network formation and stability. Optimization also increased the total carbohydrate content of SH2 in CDM to 516.45% ± 0.05%, whereas SDM yielded only 128.07% ± 0.03%, compared to 301.48% ± 0.20% under non‐optimized conditions (Allaith et al. 2022). The reduced yield in SDM may be due to osmotic stress induced by the presence of multiple sugar types, as discussed in Section 3.2.1. Similarly, Imran et al. (2016) reported enhanced carbohydrate content (95.45% and 92.35%) in EPS extracted from *L. plantarum* NTMI05 and NTMI20 under optimal conditions, with no detectable protein content. Under optimized conditions, purification steps such as heat treatment and the addition of 15% trichloroacetic acid (TCA) significantly reduced protein contamination, as also noted by Notararigo et al. (2013). In this study, SH2 grown in CDM and SDM showed reduced total protein contents (72.81% ± 0.02% and 91.40% ± 0.03%, respectively) compared to 150.60% ± 0.02% under non‐optimized conditions, reflecting improved purity post‐optimization. However, protein precipitation was not complete, possibly due to the limited 2‐h settling time intended to avoid EPS degradation. This is consistent with Oleksy‐Sobczak and Klewicka (2020), who applied prolonged heat treatment (100°C for 15 h) followed by 15% TCA to effectively remove protein contaminants. The use of either heating, TCA, or both has been widely reported as an effective EPS purification strategy under optimal extraction conditions (Suryawanshi et al. 2019; Zhao et al. 2020; Manochai et al. 2014).

**TABLE 6:** The analysis of EPS extracted from *L. fermentum* SH2 under optimal conditions compared to the non‐optimal conditions.

SH2 under different conditions	EPS mg/100 mL	Total carbohydrate %	Total protein %	β‐glucans %
SH2 (B6)	481 ± 1.00^b^	301.48 ± 0.20^b^	150.60 ± 0.02^a^	5.56 ± 0.01^c^
SH2 in SDM	117.25 ± 1.00^c^	128.07 ± 0.03^c^	91.40 ± 0.03^b^	50.823 ± 0.03^b^
SH2 in CDM	950.65 ± 3.00^a^	516.45 ± 0.05^a^	72.81 ± 0.02^c^	412.116 ± 0.01^a^

*Note:* SH2: *Limosilactobacillus fermentum* that symbolized by (B6) under non‐optimization conditions based on our former study; SH2 in SDM: the actual value for EPS production after optimal conditions in Semi Define Media; SH2 in CDM: the actual value for EPS production after optimal conditions in Chemical Define Media. a, b, c: Small letters refer to significant differences (*p* < 0.05) between the means.

**FIGURE 4:**
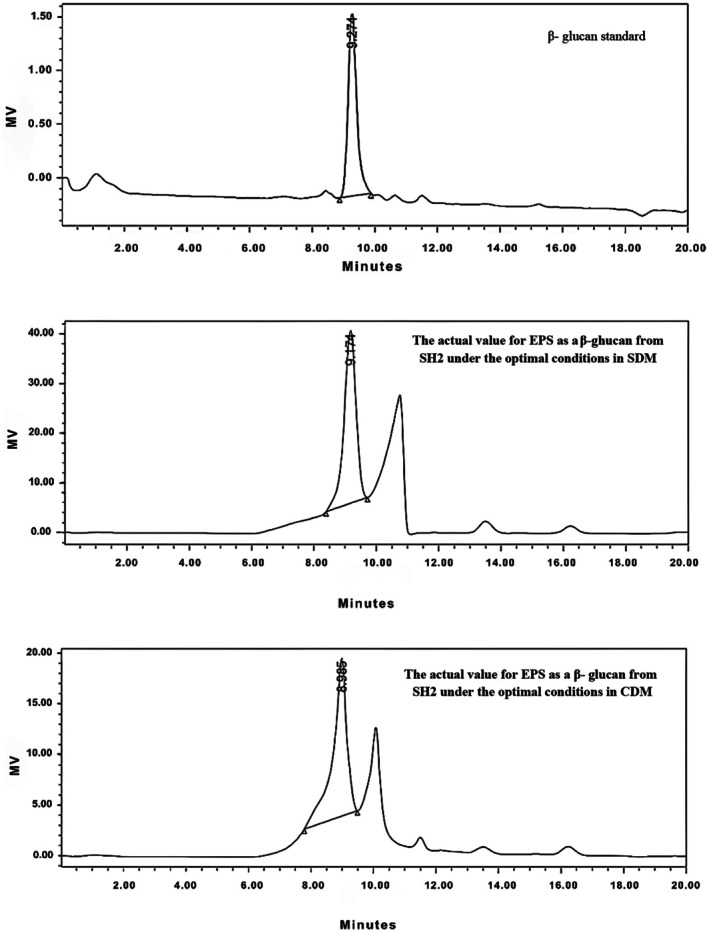
The actual value for EPS as a β‐glucan from *L. fermentum* SH2 under the optimal conditions in SDM and CDM and standard (β‐glucan).

Table 6 and Figure 4 present both quantitative and qualitative analyses of β‐glucan content in EPS extracted from *Lactobacillus fermentum* SH2 cultivated in semi‐defined media (SDM) and chemically defined media (CDM), compared to SH2 under non‐optimal conditions and a β‐glucan standard. The highest β‐glucan concentration was observed in SH2 cultured in CDM, reaching 412.116% ± 0.01%, whereas SDM‐grown SH2 showed a markedly lower content of 50.823% ± 0.03%, and SH2 under non‐optimal conditions exhibited the lowest value at 5.56% ± 0.01%. The standard curve for β‐glucan demonstrated high reliability with an *R* ^2^ of 0.992 (Allaith et al. 2022). These results indicate that fermentation under optimized conditions significantly enhances β‐glucan content in EPS, particularly when molasses is used as a carbon source in CDM. Consistent with previous findings, the retention times obtained by HPLC for SH2 β‐glucan in SDM and CDM (9.174 and 8.985 min, respectively) closely matched those of the β‐glucan standard (9.274 min) and SH2 reference (9.014 min) reported by Allaith et al. (2022). This alignment confirms the identity of the β‐glucan in the EPS samples. Comparable retention times have been reported by Abd El Ghany et al. (2016), who identified β‐glucan in EPS from *Pediococcus parvulus* F1030 with a standard retention time of 9.024 min using HPLC analysis.

### 3.4 The Bioactivity Characterization of β‐Glucan

#### 3.4.1 The Antioxidant Properties of β‐Glucan by Measuring Its Ability to Scavenge DPPH Radicals

**FIGURE 5:**
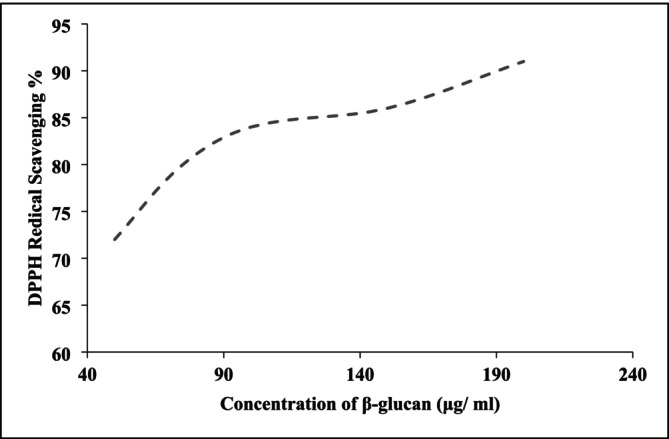
The potential to reduce DPPH at various β‐glucan concentrations that were isolated from *L. fermentum* SH2.

Figure 5 illustrates the DPPH radical scavenging activity (%) of β‐glucan extracted from *L. fermentum* SH2. The antioxidant capacity showed a significant concentration‐dependent increase, with scavenging percentages of 72% ± 0.58%, 80% ± 0.57%, 84% ± 0.58%, 86% ± 0.55%, and 91% ± 0.56% at β‐glucan concentrations of 50, 75, 100, 150, and 200 μg/mL, respectively. The highest antioxidant activity was observed at 200 μg/mL, reaching 91%. These results confirm the effective DPPH radical scavenging potential of β‐glucan derived from LAB. Our findings are consistent with those reported by Abd El Ghany et al. (2016), who observed a maximum antioxidant activity of 60% at 100 μg/mL β‐glucan extracted from *Pediococcus parvulus* .

At a concentration of 500 mg/mL, exopolysaccharides (EPS) from *Lactobacillus plantarum* strains NTMI05 and NTMI20 demonstrated strong antioxidant activity, with DPPH radical scavenging rates of 96.62% and 91.86%, respectively. This antioxidant potential is attributed to functional groups, such as hydroxyl moieties, present in both EPS and bacterial cells, which can donate electrons to neutralize free radicals or interrupt radical chain reactions (Imran et al. 2016; Shen et al. 2013). Numerous in vitro studies have further elucidated the antioxidant mechanisms of EPS derived from lactic acid bacteria (LAB), predominantly isolated from food sources. These mechanisms include scavenging reactive oxygen species (ROS) such as superoxide anions and hydrogen peroxide, inhibition of lipid peroxidation, metal ion chelation, and the modulation of enzymatic and non‐enzymatic antioxidant defenses (Zhang et al. 2013; Trabelsi et al. 2017).

#### 3.4.2 Cytotoxicity *Activity of β‐Glucan*

**FIGURE 6:**
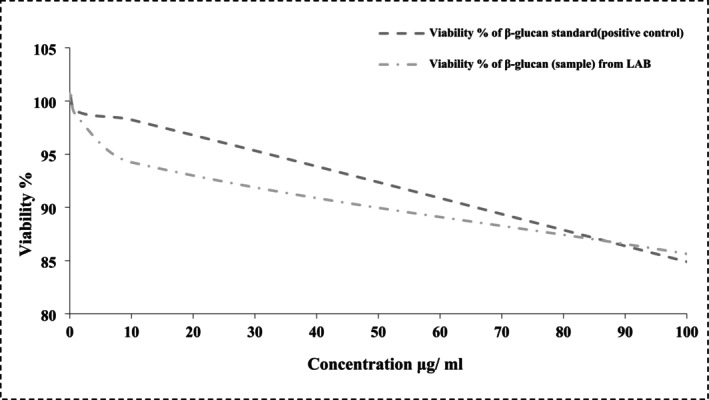
The impact of various concentrations of β‐glucan (β‐glucan standard as a positive control; β‐glucan sample from LAB) on the viability (%) of colorectal cancer (Caco‐2) cells.

The in vitro effects of β‐glucan (both standard and sample) on Caco‐2 cell viability were evaluated at five concentrations (0.01, 0.1, 1.0, 10, and 100 μg/mL) using a tissue culture microplate assay, as summarized in Table 7 and illustrated in Figure 6. Significant differences in cell viability percentages were observed, with the β‐glucan sample exhibiting a maximum viability of 85.64% ± 4.48% at 100 μg/mL, slightly higher than the positive control's 84.89% ± 2.92% at the same concentration. Notably, at 1.0 and 10 μg/mL, β‐glucan extracted from LAB demonstrated a more pronounced inhibitory effect on Caco‐2 cell proliferation, with viability values of 98.67% ± 3.32% and 94.23% ± 0.64%, respectively, compared to the standard β‐glucan (positive control) values of 99.06% ± 3.40% and 98.23% ± 3.43% at corresponding concentrations. These results indicate differential bioactivity between the β‐glucan sample and the standard across the tested concentrations.

**TABLE 7:** The different concentrations of β‐glucan (standard as a positive control, and sample from LAB) on the viability (%) of colorectal cancer (CaCo‐2) cells.

Concentration μg/mL	Viability % of β‐glucan standard (positive control)	Viability % of β‐glucan (sample) from LAB
0	100^a^	100^a^
0.01	100.975 ± 1.60^a^	101.077 ± 2.38^a^
0.1	100.855 ± 1.17^a^	100.918 ± 1.65^a^
1	99.063 ± 3.40^a^	98.669 ± 3.32^ab^
10	98.233 ± 3.43^a^	94.234 ± 0.64^b^
100	84.890 ± 2.92^b^	85.636 ± 4.48^c^

**FIGURE 7:**
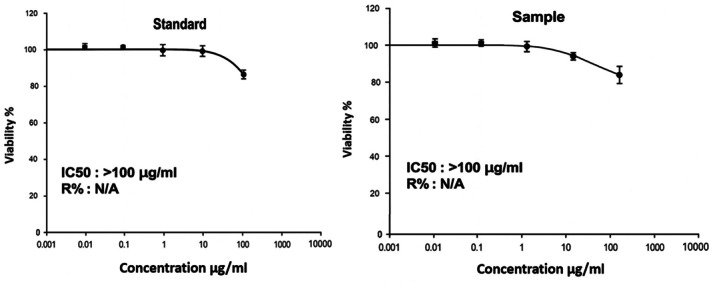
The IC_50_ response curve for the fatal concentration at > 100 μg/mL of β‐glucan (standard and sample).

**FIGURE 8:**
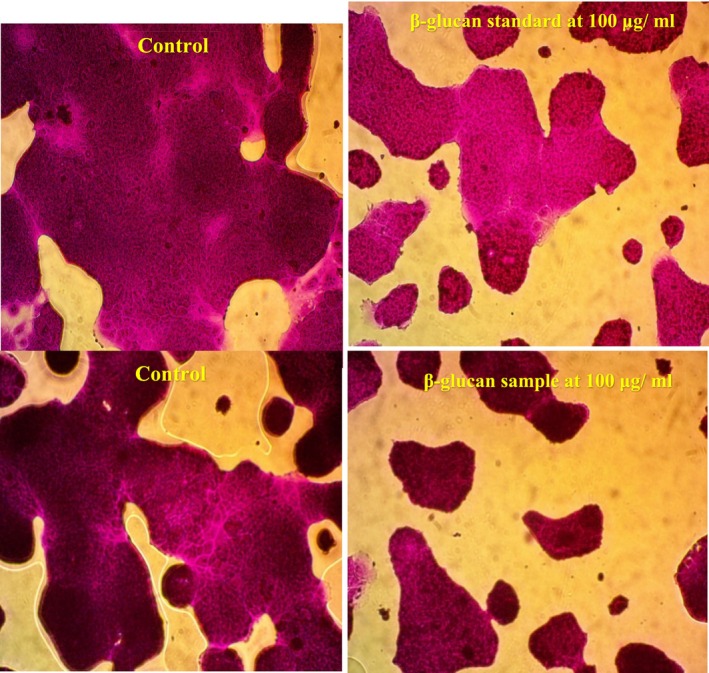
The fatal concentration at > 100 μg/mL β‐glucan (standard and sample) led to 50% of Caco‐2 cells dying.

Figures 7 and 8 illustrate the IC₅₀ values representing the concentration of β‐glucan (both standard and sample) required to induce 50% cytotoxicity in Caco‐2 cells after 27 h of exposure, with effective concentrations exceeding 100 μg/mL. Notably, β‐glucan extracted from LAB exhibited significantly higher inhibitory effects on Caco‐2 cell proliferation compared to the β‐glucan standard (positive control), as shown in Figure 8. These findings align with previous reports demonstrating the antitumor potential of bacterial EPS. For instance, Abd El Ghany et al. (2016) documented that β‐glucan from *Pediococcus parvulus* inhibited Ehrlich Ascites Carcinoma cell growth by up to 85% at 700 mg/mL. Similarly, EPS from *Lactobacillus plantarum* 70,810 significantly suppressed the proliferation of HepG‐2, BGC‐823, and HT‐29 tumor cell lines (Wang et al. 2014). Additionally, *Lactobacillus acidophilus* EPSs downregulated genes involved in angiogenesis and tumor survival in colon cancer models (Deepak, Ramachandran, et al. 2016; Deepak, Kumar Pandian, et al. 2016). Moreover, silver nanoparticles synthesized using EPS from *Levilactobacillus brevis* MSR10 (AgNPs) markedly reduced HT‐29 cell viability (Rajoka et al. 2020). EPS from lactobacilli has also been shown to induce apoptosis in HT‐29 cells, confirmed by changes in apoptotic markers via qPCR and Western blot analyses (Tukenmez et al. 2019). Collectively, these studies support the potent anticancer effects of LAB‐derived β‐glucans and EPS.

#### 3.4.3 Antibacterial Activity of Extracted β‐Glucan Against Some Pathogenic Bacteria

**FIGURE 9:**
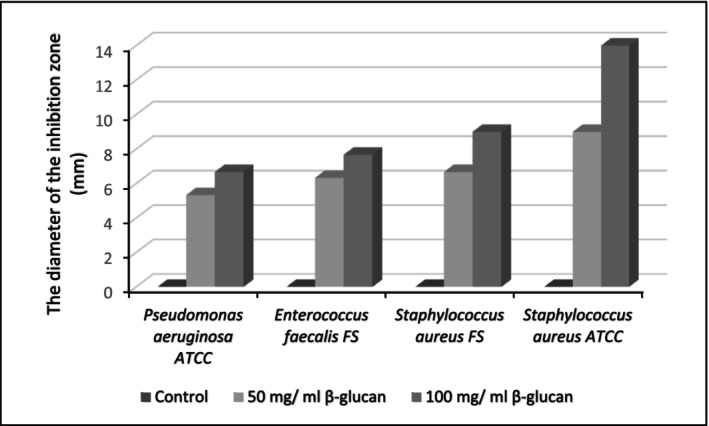
Antibacterial activity of β‐glucan in different concentrations against four pathogenic bacteria, which was estimated by the inhibition zone (mm) minus the diameter of the well.

Figure 9 illustrates the antibacterial activity of β‐glucan at two concentrations (50 and 100 mg/mL) against four pathogenic bacteria: *Pseudomonas aeruginosa* ATCC, *Enterococcus faecalis* FS, *Staphylococcus aureus* FS, and *Staphylococcus aureus* ATCC. Significant differences were observed between the β‐glucan concentrations and the resulting inhibition zones (measured in mm, excluding the well diameter). At 50 mg/mL, inhibition zones measured (5.33 ± 0.58, 6.33 ± 0.58, 6.67 ± 0.58, and 9.00 ± 1.00) mm, respectively, while the control (distilled water) showed no inhibition. Increasing the β‐glucan concentration to 100 mg/mL significantly enhanced antibacterial effects, with inhibition zones increasing to (6.67 ± 0.58, 7.67 ± 0.58, 9.00 ± 1.00, and 14.00 ± 1.00) mm, respectively. The highest inhibition was observed against *Staphylococcus aureus* ATCC at 100 mg/mL, with a 14 mm zone. These results align with previous findings by Abd El Ghany et al. (2016), who reported a maximum inhibition zone of 24 mm against *E. coli* at 200 μg/mL β‐glucan extracted from *Pediococcus parvulus* .

In vitro studies suggest that the antimicrobial activity of exopolysaccharides (EPS) produced by lactic acid bacteria (LAB) primarily stems from their ability to inhibit biofilm formation by pathogenic bacteria through disruption of cellular integrity and quorum sensing (Spanò et al. 2016; Xing et al. 2009). Biofilm formation enhances pathogenic bacteria's environmental resilience, contributing to persistent infections and increased antibiotic resistance (Rajoka et al. 2018). LAB‐derived EPS have demonstrated significant antibiofilm effects against pathogens such as *Enterococcus faecalis* , *Bacillus cereus* , and *Pseudomonas aeruginosa* (Sarikaya et al. 2017). For instance, EPS from *Lactobacillus paracasei* M7 inhibited *E. faecalis* (64.27%), *Bacillus subtilis* (63.84%), *Bacillus cereus* (62.89%), *Staphylococcus aureus* (61.45%), *Klebsiella* spp. (59.42%), and *P. aeruginosa* (58.88%) (Bhat and Bajaj 2019). Mechanistically, EPS may prevent early bacterial auto‐aggregation by interfering with cell–cell communication or compromising membrane integrity in a dose‐dependent manner (Kanmani et al. 2013; Jiang et al. 2011). Furthermore, EPS from *Lactobacillus fermentum* UCO‐979C reduced *Helicobacter pylori* adhesion by approximately 30% in both in vitro and in vivo models, concurrently enhancing host immune responses against infection (Garcia‐Castillo et al. 2020).

## 4 Conclusion

This study successfully optimized β‐glucan production by *Limosilactobacillus fermentum* SH2 (MW897962) using statistical modeling through Central Composite Design (CCD), demonstrating the superior performance of chemically defined media supplemented with molasses. The findings confirmed that molasses, as a cost‐effective carbon source, significantly enhanced EPS and β‐glucan yields, indicating its industrial scalability. The produced β‐glucan exhibited promising bioactivity, including strong antioxidant capacity, moderate cytotoxic activity against Caco‐2 cancer cells, and notable antibacterial effects against common pathogens, suggesting its multifunctional health‐promoting potential. These outcomes underscore the value of *L. fermentum* SH2 as a biotechnological candidate for producing functional polysaccharides with potential applications in nutraceuticals, pharmaceuticals, and food industries. Moreover, the study highlights the feasibility of using agro‐industrial byproducts, like molasses, to reduce production costs and support sustainable bioprocessing. Future research should focus on scaling up the production process, characterizing the structural and rheological properties of the purified β‐glucan, and conducting in vivo studies to validate its health benefits. Further exploration into its immunomodulatory and anti‐inflammatory effects, as well as its potential role in microbiome modulation, would provide deeper insights into its mechanisms of action and broaden its applications in functional food development and preventive healthcare.

## Author Contributions

**Tarek Gamal Abedelmaksoud:** conceptualization (equal), methodology (equal), project administration (equal), validation (equal), writing – review and editing (equal). **Shatha A. Allaith:** data curation (equal), formal analysis (equal), investigation (equal), methodology (equal), writing – original draft (equal). **Ammar B. Altemimi:** conceptualization (equal), methodology (equal), validation (equal), writing – review and editing (equal). **Mohamed E. Abdel‐aziz:** formal analysis (equal), methodology (equal), validation (equal), writing – original draft (equal). **Zaid Akram Thabit:** formal analysis (equal), investigation (equal), software (equal), writing – original draft (equal). **Mohammad Ali Hesarinejad:** conceptualization (equal), validation (equal), writing – review and editing (equal). **Reda Mahgoub Mohamed:** investigation (equal), methodology (equal), software (equal), validation (equal), writing – review and editing (equal).

## Conflicts of Interest

The authors declare no conflicts of interest.

## Data Availability

Data are contained within the article.
